# Supplementary material for: Planning ahead for research participation: survey of public and professional stakeholders’ views about the acceptability and feasibility of advance research planning
Source: BMC Med Ethics. 2023 Sep 9;24:70. doi: 10.1186/s12910-023-00948-3 (PMC10492324; doi:10.1186/s12910-023-00948-3)
Supplement: Supplementary file 3 — Supplementary Material 3 [file 12910_2023_948_MOESM3_ESM.docx]

## Supplementary File 2

*Figure S1. Public stakeholders’ views about timing of advance research planning*

*Figure S2. Professional stakeholders’ views about timing of advance research planning*

*Figure S3. Public stakeholders’ views about the content of advance research planning*

*Figure S4. Professional stakeholders’ views about the content of advance research planning*

*Figure S5. Public stakeholders’ views about how advance research planning should inform decisions about research*

*Figure S6. Professional stakeholders’ views about how advance research planning should inform decisions about research*

*Figure S7. Public stakeholders’ views about what form advance research planning should take*

*Figure S8. Professional stakeholders’ views about what form advance research planning should take*

*Figure S9. Public stakeholders’ views about who should be involved in advance research planning*

*Figure S10. Professional stakeholders’ views about who should be involved in advance research planning*
